# Rapid-Response and Highly Sensitive Boronate Derivative-Based Fluorescence Probe for Detecting H_2_O_2_ in Living Cells

**DOI:** 10.1155/2019/5174764

**Published:** 2019-05-02

**Authors:** Muthusamy Selvaraj, Kanagaraj Rajalakshmi, Yun-Sik Nam, Yeonhee Lee, Byoung Chan Kim, Sung Jin Pai, Sang Soo Han, Kang-Bong Lee

**Affiliations:** ^1^National Agenda Research Division, Korea Institute of Science & Technology, Hwarang-ro 14-gil 5 Seongbuk-gu, Seoul 02792, Republic of Korea; ^2^Advanced Analysis Center, Korea Institute of Science & Technology, Hwarang-ro 14-gil 5 Seongbuk-gu, Seoul 02792, Republic of Korea; ^3^Computational Science Center, Korea Institute of Science & Technology, Hwarang-ro 14-gil 5 Seongbuk-gu, Seoul 02792, Republic of Korea

## Abstract

Intracellular H_2_O_2_ monitoring is important and has driven researchers to pursue advancements for the rapid identification of H_2_O_2_, since H_2_O_2_ is short-lived in cell lines. An arylboronate derivative has been investigated as a chemospecific fluorescence recognition agent for H_2_O_2_. Triphenylimidazoleoxadiazolephenyl (TPIOP) boronate was contrived as a novel candidate for the rapid and sensitive recognition of H_2_O_2_. The probe was conjugated using the TPIOP functional group acting as an excellent fluorescent enhancer. The TPIOP group stimulated the polarization of C–B bond due to its extended *π*-conjugation, which included heteroatoms, and induced the production of rapid signal because of the highly polar C–B bond along with the corresponding boronate unit. While H_2_O_2_ reacts with TPIOP boronate, its nucleophilic addition to the boron generates a charged tetrahedral boronate complex, and then the C–B bond migrates toward one of the electrophilic peroxide oxygen atoms. The resulting boronate ester is then hydrolyzed by water into a phenol, which significantly enhances fluorescence through aggregation-induced emission. The TPIOP boronate probe responded to H_2_O_2_ rapidly, within 2 min, and exhibited high sensitivity with a limit of detection of 8 nM and a 1000-fold selectivity in the presence of other reactive oxygen species. Therefore, the developed TPIOP boronate chemodosimeter was successfully utilized to visualize and quantify intracellular H_2_O_2_ from human breast cancer (MCF-7) cells, as well as gaseous and aqueous H_2_O_2_ from environmental samples using Whatman paper strips coated with TPIOP boronate.

## 1. Introduction

Hydrogen peroxide (H_2_O_2_) is a small yet important reactive oxygen species (ROS), which is present in biological systems, and exerts wide physiological and pathological effects [1]. Abnormal levels of H_2_O_2_ in the human body can cause long-term damage to cells and organs and lead to significant neurodegeneration, oxidative stress, and cancer [2]. Thus, the far-ranging impingements of H_2_O_2_ homeostasis encouraged scientists to construct sensitive, rapid response, and selective and accurate sensors for detecting H_2_O_2_.

H_2_O_2_ presents a unique inherent conflict of reactivity over the other ROS, because most of the other ROS are operated by one electron transfer pathway. Moreover, H_2_O_2_ possesses amphiphilic reactivity; its labile O–O bond allows it to react as a two-electron electrophilic oxidant, whereas H_2_O_2_ can also be a good nucleophile due to the *α*-effect of the adjacent nonbonding orbitals on its oxygen atoms [3, 4]. Boronates present unique abilities for detecting amphiphilic substances and exhibit chemoselectivity for H_2_O_2_, while the aryl derivative bound to the boron atom converts to phenol when reacting with H_2_O_2_ [3, 4].

Although numerous aryl derivatives conjugated with boronate have been developed as H_2_O_2_ chemosensors, their slow response times cause difficulties for tracking H_2_O_2_ in situ [5–30]. Since H_2_O_2_ has a short half-life, the slow responses of H_2_O_2_ chemosensors caused researchers to question the accuracy of the measured concentrations of H_2_O_2_ from in situ cell lines and the environment. Therefore, designing a novel molecule which would facilitate the fast detection of H_2_O_2_ has been a challenge. Zhang et al. recently reported that the polarity of the C–B bond in boronate represented the key to achieving fast and sensitive recognition of H_2_O_2_ [5–7]. They determined that the extended conjugation of the *π*-electron system of tetraphenylethylene (TPE) combined with boronate compound could enhance the polarity of C–B bond, thus achieving rapid monitoring of H_2_O_2_ through aggregation-induced emission (AIE) [5–7]. This finding opened a new window toward designing a fluorogenic probe for H_2_O_2_ sensing through significantly increasing the C–B bond polarity. Incorporating heteroatoms into a fluorogenic probe using new structural design strategies for synthesizing intensive AIE luminogens caused large electron perturbations and increased the emission intensity of luminogens [31]. For example, imidazole rings present lone pairs of electron-rich nitrogen atoms, which can induce polarization by intramolecular charge transfer and have been widely exploited in the fields of biology and fluorescent sensors [32]. Many researches have recently been focusing on derivatives of phenylimidazole chemosensors based on their enhanced fluorescence properties. For example, Takagi et al. synthesized fused *π*-conjugated diphenylimidazole derivatives, which exhibited superior optical properties [33]. Based on these findings, we designed an imidazole containing boronate, which included a triphenyl group in the imidazole ring bridged with oxadiazolepheylboronate, and utilized it for H_2_O_2_ detection. The additional oxadiazole ring was introduced to enhance the electron affinity associated with the electron transporting properties of the highly photoluminescent compound [34].

The newly synthesized triphenylimidazoleoxadiazolephenyl (TPIOP) boronate chemosensor was tested as a fast-response and highly sensitive chemosensor. The high degree of *π*-conjugation created an excellent electrostatic potential on the carbon atom bound to the boron atom. The boronate group acted as a recognition unit, and the extended triphenylimidazoleoxadiazole moiety significantly amplified the inherent fluorescence of the chemosensor compared to that of the earlier chemodosimeter containing a simple phenyl unit. As expected, the TPIOP boronate probe responded rapidly to H_2_O_2_, within 2 min of coming into contact with H_2_O_2_. When the probe was triggered using H_2_O_2_, the weakly fluorescent TPIOP boronate converted into triphenylimidazoleoxadiazolephenol (TPIOP-OH), which exhibited much stronger fluorescence, and the enhanced emission intensity was caused by the mechanistic pathway of AIE. The limit of detection (LOD) of H_2_O_2_ for the TPIOP boronate probe was approximately 8 nM. Also, H_2_O_2_ can be visually detected by exposing Whatman paper coated with TPIOP boronate to UV radiation. This TPIOP boronate H_2_O_2_ chemodosimeter exhibited rapid response, high chemoselectivity, high sensitivity, and low cytotoxicity, which rendered it useful for detecting H_2_O_2_ in MCF-7 (human breast cancer) cell lines.

## 2. Materials and Methods

### 2.1. Chemicals and Reagents

4-Cyanobenzaldehyde, benzil, 4-bromobenzoic acid, bis(pinacolato)diboron, 1,1′-carbonyldiimidazole (CDI), [1,1′-bis(diphenylphosphino)ferrocene]dichloropalladium(II)dichloride (PdCl_2_ (dppf)), H_2_O_2_, potassium super-oxide (KO_2_), t-butyl hydroperoxide (TBHP), m-chloroperoxybenzoic acid (mCPBA), hypochlorous acid (HOCl), HEPES buffer solution, Whatman papers, Dulbecco's phosphate-buffered saline (DBS), Roswell Park Memorial Institute (RPMI) medium, fetal bovine serum (FBS), penicillin, streptomycin, and 3-(4,5-dimethylthiazol-2-yl)-2,5-diphenyltetrazolium bromide (MTT) were purchased from Sigma-Aldrich (St. Louis, MO, USA). Ammonium acetate, acetic acid, ethanol, hydroxylamine hydrochloride, sodium hydroxide, ethyl acetate, hexane, sodium bicarbonate, dimethylformamide (DMF), dimethyl sulfoxide (DMSO), 1,4-dioxane, and potassium acetate were purchased from Daejung Chemicals (Siheungsi, Kyunggido, Republic of Korea). Sodium nitrate (NaNO_3_^−^), potassium perchlorate (KClO_4_^−^), sodium sulfate (Na_2_SO_4_), sodium nitrite (NaNO_2_), and benzoyl peroxide (BPO) were obtained from AccuStandard (New Haven, CT, USA). The MCF-7 cells were purchased from the Korean Cell Line Bank (Seoul, Republic of Korea). All reagents were used as received without further purification.

### 2.2. Instrumentation

All UV-Vis absorption spectra were measured in the 300–800 nm range, using polystyrene cells of 1 mm path lengths on an S-3100 spectrophotometer (Sinco, Seoul, Republic of Korea). Fluorescence spectra were recorded using a LS-45 luminescence spectrometer (PerkinElmer, Waltham, MA, US). Both ^1^H and ^13^C nuclear magnetic resonance (NMR) spectra were recorded using a Bruker Avance 600 MHz spectrometer (Billerica, MA, US). The mass spectra were obtained in positive mode using a Synapt G2 high-resolution mass spectrometer (HR-MS) (Waters, Milford, MA, US). The pH of the solution was adjusted using a HI 2210 pH meter (Hanna Instruments, Woonsocket, RI, USA). Fluorescence images were acquired using a LSM 700 confocal laser scanning microscope (Carl Zeiss, Jena, Germany) equipped with a 63× oil immersion objective lens and a diode laser as light source. Cytotoxicity tests were performed using a Spectramax M2 microplate reader (Molecular Devices, Sunnyvale, CA, USA).

### 2.3. Synthesis of TPIOP Boronate

#### 2.3.1. Preparation of 4-(4,5-Diphenyl-1H-imidazol-2-yl)benzonitrile

**Scheme 1:**
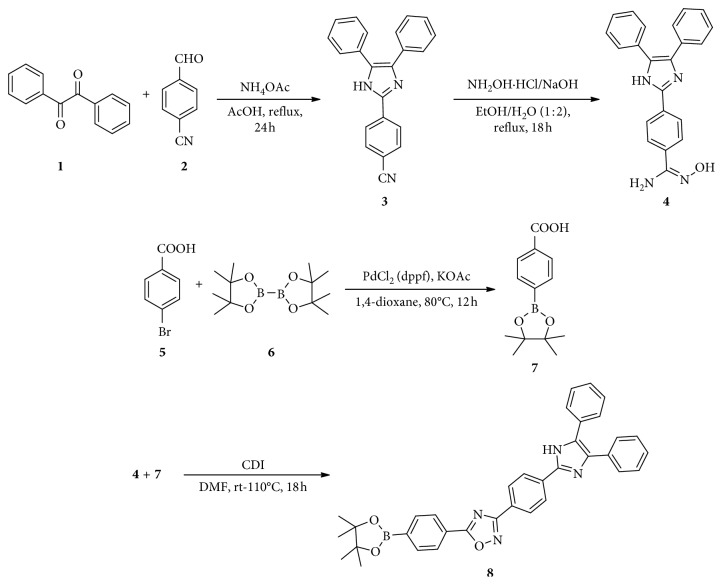
Synthesis of TPIOP boronate.

A solution of 4-cyanobenzaldehyde ((**2**) in Scheme 1) (1.80 g, 13.73 mM), benzil ((**1**) in Scheme 1) (2.90 g, 14.1 mM), and ammonium acetate (8.50 g, 110.39 mM) in acetic acid (50 mL) was refluxed for 24 h under N_2_ flow. After cooling, the solution was poured into ice-cooled water to precipitate the product. The yellow solid was collected and dried to form 4-(4,5-diphenyl-1H-imidazol-2-yl)benzonitrile ((**3**) in Scheme 1) (4.20 g) with 95% yield.

^1^H NMR (400 MHz, DMSO) *δ* 13.01 (brs, 1H), 8.25 (d, *J* = 8.4 Hz, 2H), 7.94 (d, *J* = 8.4 Hz, 2H), 7.53 (d, *J* = 7.2 Hz, 4H), 7.45–7.29 (m, 6H). ^13^C NMR (100 MHz, DMSO) *δ* 141.2, 134.8, 133.2, 130.1, 130.0, 129.0, 128.8, 128.5, 126.0, 119.4, 110.6.

#### 2.3.2. Preparation of *N*′-Hydroxy-4-(4,5-Diphenyl-1H-imidazol-2-yl)benzamidine

Hydroxylamine hydrochloride (NH_2_OH·HCl) (18.8 mmol, 1.31 g) and sodium hydroxide (18.8 mmol, 752 mg) were added to a solution of benzonitrile ((**3**) in Scheme 1) (17.1 mM, 5.50 g) in ethanol (5 mL), and then water (10 mL) was sequentially added to the solution for 20 min at 0°C. Then, the resulting mixture was allowed to reflux while stirring for 18 h. The pH of the solution was adjusted to 2 using 1 N HCl, and the aqueous phase was washed using ethyl acetate. Upon cooling (0°C) and neutralization with sodium bicarbonate, a precipitate formed, which was subsequently filtered, washed, and dried to obtain pure *N*′-hydroxy-4-(4,5-diphenyl-1H-imidazol-2-yl)benzamidine ((**4**) in Scheme 1) (5.30 g) with 87% yield.

^1^H NMR (400 MHz, DMSO) *δ* 12.73 (brs, 1H), 9.71 (s, 1H), 8.07 (d, *J* = 8.4 Hz, 2H), 7.77 (d, *J* = 8.4 Hz, 2H), 7.55 (d, *J* = 7.3 Hz, 4H), 7.46–7.20 (m, 6H), 5.84 (s, 2H). ^13^C NMR (100 MHz, DMSO) *δ* 151.2, 145.8, 133.4, 131.3, 128.9, 127.7, 127.5, 127.2, 126.1, 125.2, 125.0.

#### 2.3.3. Preparation of 4-Carboxyphenyl Boronate (CPB)

Bis(pinacolato)diboron ((**6**) in Scheme 1) (5.47 mM, 1.40 g), PdCl_2_(dppf) (3 mol%, 0.49 mM, 109.00 mg), and potassium acetate (14.93 mM, 1.50 g) were added to a mixture of benzoic acid ((**5**) in Scheme 1) (4.98 mM, 1.00 g) in dry 1,4-dioxane (10 ml). Then, resulting mixture was degassed three times using N_2_ gas and was stirred at 80°C for 12 h. The reaction mixture was filtered through celite and concentrated in vacuo. The brown CPB solid (IUPAC name: 4-(4,4,5,5-tetramethyl-1,3,2-dioxaborolan-2-yl)benzoic acid) (963.70 mg) was obtained with a good 78% yield.

^1^H NMR (400 MHz, DMSO) *δ* 13.04 (brs, 1H), 7.94 (d, *J* = 8.0 Hz, 2H), 7.77 (d, *J* = 8.0 Hz, 2H), 1.29 (s, 12H). ^13^C NMR (100 MHz, DMSO) *δ* 168.1, 135.3, 134.0, 132.4, 129.1, 85.0, 25.6.

#### 2.3.4. Preparation of TPIOP Boronate

Carbonyl diimidazole (0.90 mM, 145.90 mg) in N_2_ was added to a solution of benzoic acid ((**7**) in Scheme 1) (0.75 mM, 186.10 mg) in dry DMF (3 ml), and the reaction mixture was stirred for 1 hr at ambient temperature. Then, *N*′-hydroxy-4-(4,5-diphenyl-1H-imidazol-2-yl)benzamidine ((**4**) in Scheme 1) (0.90 mM, 320 mg) was slowly added to the above mix, and the reaction mixture was heated at 110°C for ∼18 h. The reaction mixture was cooled to 25°C and poured into ice-cold water (25 mL) and extracted using methylene chloride. The combined organic phase was washed with brine, dried over anhydrous sodium sulfate, filtered, and concentrated in vacuo. The obtained residue was purified utilizing column chromatography using silica gel to generate pure TPIOP boronate (IUPAC name: 5-(4-(4,4,5,5-tetramethyl-1,3,2-dioxaborolan-2-yl)phenyl)-3-(4-(4,5-diphenyl-1H-imidazol-2-yl)phenyl)-1,2,4-oxadiazole) ((**8**) in Scheme 1) (369.60 mg) as a brown solid substance with excellent 87% yield.

^1^H NMR (400 MHz, DMSO) *δ* 12.94 (brs, 1H), 8.92 (dd, *J* = 1.6, 4.4 Hz, 2H), 8.31 (d, *J* = 8.5 Hz, 2H), 8.20 (d, *J* = 8.5 Hz, 2H), 8.12 (dd, *J* = 1.6, 4.4 Hz, 2H), 7.56 (d, *J* = 7.2 Hz, 2H), 7.52 (d, *J* = 7.2 Hz, 2H), 7.47–7.23 (m, 6H), 1.22 (s, 12H) (Figure S1A).^13^C NMR (100 MHz, DMSO) *δ* 168.2, 163.5, 154.1, 151.4, 144.1, 133.2, 132.5, 131.1, 129.7, 127.5, 124.2, 123.7, 123.5, 81.3, 25.6 (Figure S2A). The *m*/*z* value was determined to be 567.46 (Figure S3A).

### 2.4. Spectroscopy Measurements

The TPIOP boronate solution was prepared using 10 mM HEPES buffer at pH 7.4 containing 2 vol% DMSO. The final concentrations of the TPIOP boronate solution used to carry out UV-Vis and fluorescence measurements were 20 and 2 *μ*M in 10 mM HEPES buffer at pH 7.4 containing 2 vol% of DMSO, respectively. In addition, 15 mM solutions of ROS such as KO_2_, NO_3_^−^, TBHP, mCPBA, HOCl, ClO_4_^−^, SO_4_^2−^, NO_2_^−^, NO_3_^−^, and BPO (benzoyl peroxide) were prepared using double distilled water.

### 2.5. Computational Methods

Quantum calculations using the density functional theory (DFT) were carried out for the abovementioned probe molecule. The generalized gradient approximation method involved the Becke three-parameter plus Lee-Yang-Parr (B3LYP) functional, while the basis set was the Pople 6-31 + G(d,p) one [33–35]. The intrinsic solvent model was considered using the c-pcm model, the dielectric constant being 78.39. All calculations were carried out using the Q-Chem 4.3 program. Graphical representation for the calculated results was obtained using the IQmol software [36, 37].

### 2.6. Paper Strip Test

For the paper strip-based detection of H_2_O_2_, Whatman paper (8 mm diameter) samples were first immersed into 1 *μ*M TPIOP boronate solution for 5 min and subsequently dried in air. The dried papers were exposed to different concentrations of H_2_O_2_ for 2 min. Then, the papers were illuminated under a UV lamp (365 nm), and fluorescence images were obtained.

### 2.7. Cell Culture, Cytotoxicity Tests, and Confocal Microscopy Imaging

The MCF-7 cells were cultured in RPMI 1640 supplemented with 10% FBS, 100 *μ*g/mL penicillin, and 100 *μ*g/mL streptomycin. The cells were maintained in an incubator at 37°C in 5% CO_2_ environments. For the cytotoxicity tests, the cells were seeded in a 96-well plate containing culture media. After overnight culture, the cells were incubated using different concentrations of TPIOP boronate. To measure the viability of the cells, 0.5 mg/mL MTT medium was added to each of the cells for 4 h and the produced formazan was dissolved in 0.1 mL DMSO and analyzed using a Spectramax microwell plate reader. The cytotoxic effects of TPIOP boronate were calculated using the following equation:

(1) $$\begin{array}{c} \text{Cell viability}\left(\%\right)=\frac{\text{OD}\left(\text{sample}\right)}{\text{OD}\left(\text{control}\right)}\times 100\%, \end{array}$$

where OD(sample) and OD(control) are the optical densities of the sample and control, respectively.

For live cells imaging, cells were seeded in 35 mm glass-bottomed dishes containing culture media. After overnight culture, the MCF-7 cells were incubated with 10 *μ*M TPIOP boronate for 30 min, washed with DBS, and incubated with 10 *μ*M H_2_O_2_ for 30 min. Fluorescence images were acquired using an LSM 700 confocal laser scanning microscope (Carl Zeiss, Jena, Germany) equipped with a 63× oil immersion objective lens and a diode laser as light source [38].

## 3. Results and Discussion

### 3.1. Spectral Studies of TPIOP Boronate

**Figure 1:**
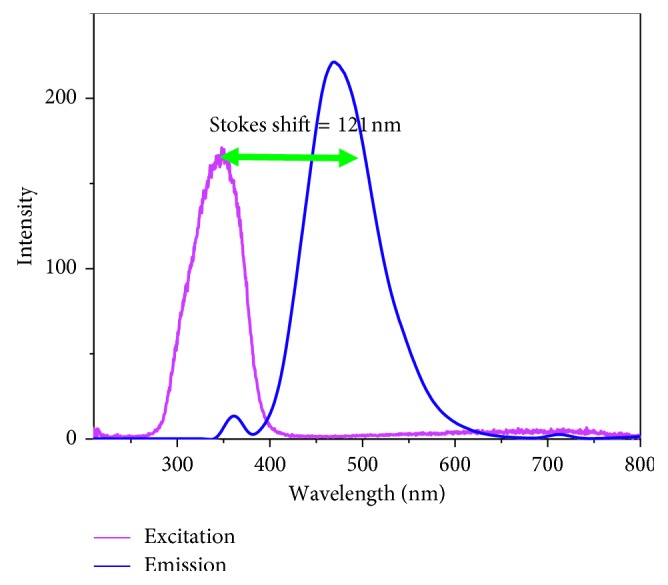
Emission (*λ*_max_ = 467 nm) and its corresponding excitation (*λ*_max_ = 346 nm) spectra obtained for 2 *μ*M TPIOP boronate in HEPES buffer solution (10 mM, pH 7.4, and 2 vol% DMSO).

**Figure 2:**
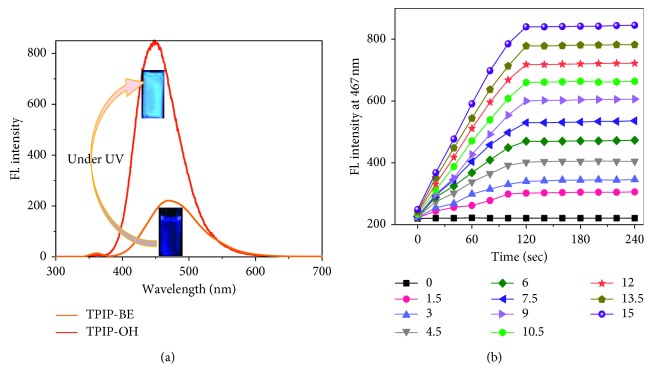
(a) Fluorescence emission spectra of the probe TPIOP boronate (1) before and (2) after reacting with H_2_O_2_ to form TPIOP-OH. (b) Time-dependent fluorescence intensity at 467 nm in 2 *μ*M TPIOP boronate using various concentrations (0 to 15 *μ*M) of H_2_O_2_ at 10 mM HEPES buffer solution (pH 7.4 and 2 vol% DMSO).

The detailed synthesis of TPIOP boronate is represented in Scheme 1. The TPIOP boronate probe was characterized using ^1^H NMR, ^13^C NMR, and HR-MS techniques (Figures S1A, S2A, and S3A, respectively). We initially evaluated the photophysical properties of the TPIOP boronate probe, and its ability to track H_2_O_2_ was investigated using HEPES buffer solution (10 mM, pH 7.4 containing 2 vol% DMSO). The optical properties of TPIOP boronate were analyzed, and the results are represented own in Figure 1. The 2 *μ*M solution of TPIOP boronate in 10 mM HEPES buffer solution (10 mM, pH 7.4 containing 2 vol% of DMSO) exhibited an excitation maximum centered at 346 nm. The TPIOP boronate probe presented weak fluorescence at 467 nm with a Stokes shift of 121 nm (Figure 1). After the probe was triggered using H_2_O_2_, a significant turn-on fluorescent enhancement was observed at 467 nm, and the fluorescence was also visually observed using an UV lamp, as shown in Figure 2(a).

**Scheme 2:**
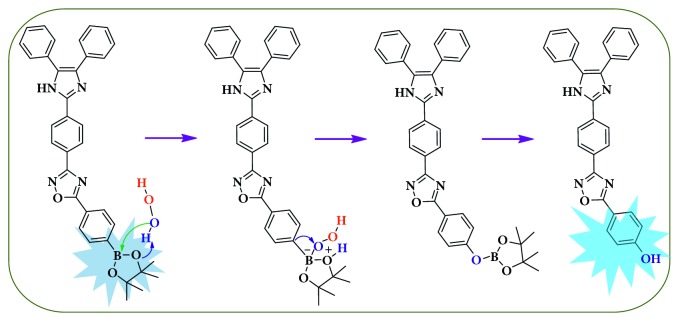
Chemical structure of TPIOP boronate and its reaction product with H_2_O_2_ (TPIOP-OH) via the proposed mechanism pathway.

The TPIOP boronate probe contained TPIOP and a boronic ester moiety in its structure. It is well known that the boronate group undergoes oxidation in the presence of H_2_O_2_, and the oxidation of boronate is caused by the enhanced nucleophilicity of H_2_O_2_ due to the *α*-effect, imparted by the adjacent nonbonding orbitals on its oxygen atoms and its weak O–O bonds. The nucleophilic addition of H_2_O_2_ to the boron atom results in a charged tetrahedral boronate complex, which subsequently undergoes a 1,2-insertion where the C–B bond migrates to one of the electrophilic peroxide oxygen atoms. The resulting borate ester is then hydrolyzed by water into phenol (Scheme 2) [6, 10, 11].

The conversion of TPIOP boronate into TPIOP-OH was supported by ^1^H NMR, ^13^C NMR, and HR-MS data (Figures S1B, S2B, and S3B, respectively). The ^1^H NMR of TPIOP boronate presented a methyl singlet peak at 1.07 ppm, which disappeared after TPIOP boronate reacted with H_2_O_2_. Then, a new peak appeared at 6.25 ppm, which was attributed to phenolic –OH proton (Figure S1B). The ^13^C NMR spectrum contained a methyl peak which appeared at 25.5 ppm and disappeared after the conversion of boronate into phenol (Figure S2B). After injecting H_2_O_2_ into the TPIOP boronate sample, a new *m*/*z* value was observed at 457.49 [M]^+^, which corresponded to the molecular weight of TPIOP-OH (Figure S3B). This demonstrated that while reacting with H_2_O_2_, TPIOP boronate was converted into TPIOP-OH (Scheme 2), and the yield was found to be 78%.

The conformational change from hydrophobic to hydrophilic form during the conversion of TPIOP boronate into TPIOP-OH led to aggregation. Intramolecular rotation associated with this conversion was restricted due to physical constraints, which blocked the nonradiative relaxation and commenced the radiative decay. Therefore, the intensity of the emission was enhanced based on an AIE mechanism.

A computational study was utilized to elucidate the characteristics of the C–B and B–O chemical bonds in TPIOP boronate. The polarities of the C–B and B–O bonds were relatively high given the electronegativities of the individual atoms (Figures S4 and S5), and the C–B bond was the most labile site for the H_2_O_2_ (Scheme 2). This was caused by extensively delocalized *π*-electrons throughout the TPIOP group. Moreover, the presence of heteroatoms increased the electron perturbation throughout TPIOP in half. Therefore, the electron density of the carbon atom bound to boron was high, which would lead to the high polarity of the C–B bond. This leads to the activation of the reaction centers during oxidation reaction of boronate in the TPIOP boronate probe, which allowed for the rapid identification of H_2_O_2_. To achieve rapid H_2_O_2_ detection using TPIOP boronate, we examined the time-dependent fluorescent kinetics, as shown in Figure 2(b). Upon adding H_2_O_2_ to TPIOP boronate, the fluorescence intensity at 467 nm increased rapidly with time and achieved its maximum within 2 mins, which intimated that TPIOP boronate was an effective fluorescent probe able to detect H_2_O_2_ very rapidly. This was due to the presence of active reaction centers (C and B) in TPIOP boronate, which were provided by the TPIOP moiety.

### 3.2. Sensitive Detection of H_2_O_2_ Using TPIOP Boronate

**Figure 3:**
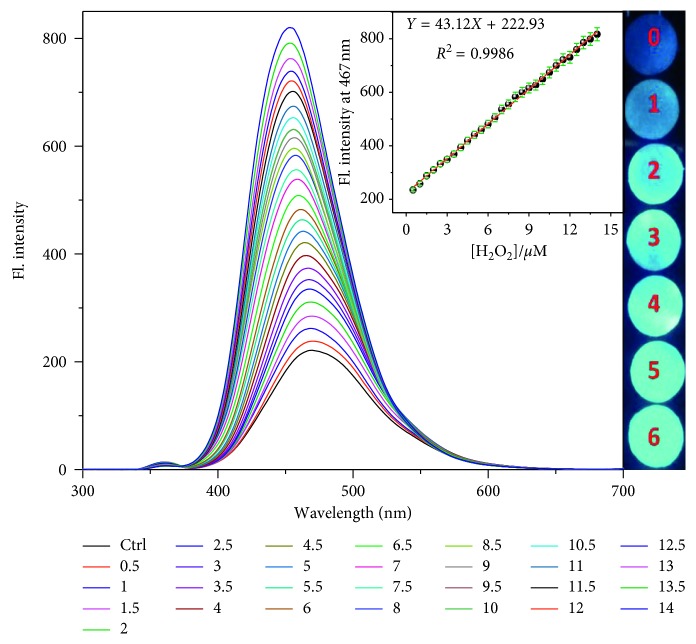
Fluorescence response of TPIOP boronate after reacting with different concentrations of H_2_O_2_ (0–13.5 *μ*M) in 10 mM HEPES buffer solution (pH 7.4 and 2 vol% DMSO). Insets: plot of fluorescent intensity at 467 nm against the concentration of H_2_O_2_ concentration and fluorescent images obtained when 2 *μ*M TPIOP boronate coated Whatman paper was exposed to different concentrations of H_2_O_2_ (0–6 *μ*M).

The initial detection ability of TPIOP boronate for H_2_O_2_ was analyzed using UV-Vis absorbance studies. We added solutions of H_2_O_2_ with concentrations ranging from 1 to 11 *μ*M to 20 *μ*M TPIOP boronate (pH 7.4, HEPES buffer solution, 2 vol% DMSO) during these experiments. The absorbance intensity at 270 nm increased linearly as the concentration of H_2_O_2_ increased from 1 to 11 *μ*M (Figure S6A). A good linearity was obtained between the concentration of H_2_O_2_ and absorbance intensity at 270 nm (*R* ^2^ = 0.9896) (Figure S6B). Sensitive detection of H_2_O_2_ was further achieved through fluorescence spectroscopy.  Figure 3 illustrates the increase in fluorescence intensity as the concentration of H_2_O_2_ increased from 0 to 13.5 *μ*M. A linear calibration curve with the regression coefficient of 0.9986 was obtained when the fluorescence intensity at 467 nm was plotted against the concentration of H_2_O_2_ (inset, Figure 3). The LOD was estimated to be 8 nM (signal to noise ratio (*S*/*N*) = 3), which was significantly lower than the reported range (Table 1). This revealed that the TPIOP boronate probe was able to detect nanomolar level concentration of H_2_O_2_.

Considering the best performance of TPIOP boronate, we also extended the study to create solid state H_2_O_2_ sensors using paper strips. We determined that depending on its concentration; H_2_O_2_ renders Whatman paper emissive (inset, Figure 3). This demonstrated that the paper strips coated with TPIOP boronate could potentially act as environmental H_2_O_2_ detector.

### 3.3. DFT Calculations on Molecular Orbitals of TPIOP Boronate

**Figure 4:**
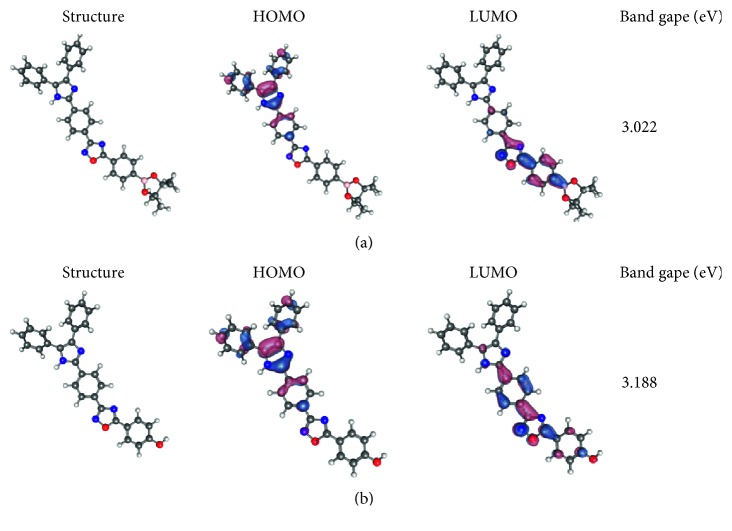
Optimized geometries of TPIOP boronate and TPIOP-OH and their HOMO-LUMO energy gaps.

To analyze the sensitivity of the abovementioned probe, the frontier molecular orbitals in TPIOP boronate were calculated and are represented in Figure 4. The splitting of the highest occupied molecular orbital (HOMO) and lowest unoccupied molecular orbital (LUMO) energy levels occurred at the opposite ends of the molecule: HOMO was located at the opposite end of the boronate group, whereas LUMO was located near the C–B bond. As can be seen from the LUMO of TPIOP boronate, *π* orbitals extended from the biphenyl moiety to the boron atom. It is well known that the mechanism for sensing H_2_O_2_ of TPIOP boronate involves the oxygen atom of H_2_O_2_ interacting with the C–B bond through a Lewis acidic reaction. The LUMO level of the probe in this reaction was lowered by HOO^−^, which facilitated the breaking of the C–B bond. When HOMO located near LUMO, the possibility of electron transitions would be high and the interaction with HOO^–^ would be obstructed by the electron-rich area near the C–B bond. The electron transitions might be hindered by the splitting of the frontier orbitals, which is responsible for the high sensitivity of TPIOP boronate for H_2_O_2_. The HOMO/LUMO transition energy was quantitatively calculated employing time-dependent DFT using optimized conformations and DFT calculations (Figure 4). It can be seen that the energy gap increased from 3.022 to 3.188 eV after TPIOP boronate reacted with H_2_O_2_. These findings coincided with the experimental data obtained using UV spectroscopy (Figure S6A).

### 3.4. Selectivity and Interference Study

**Figure 5:**
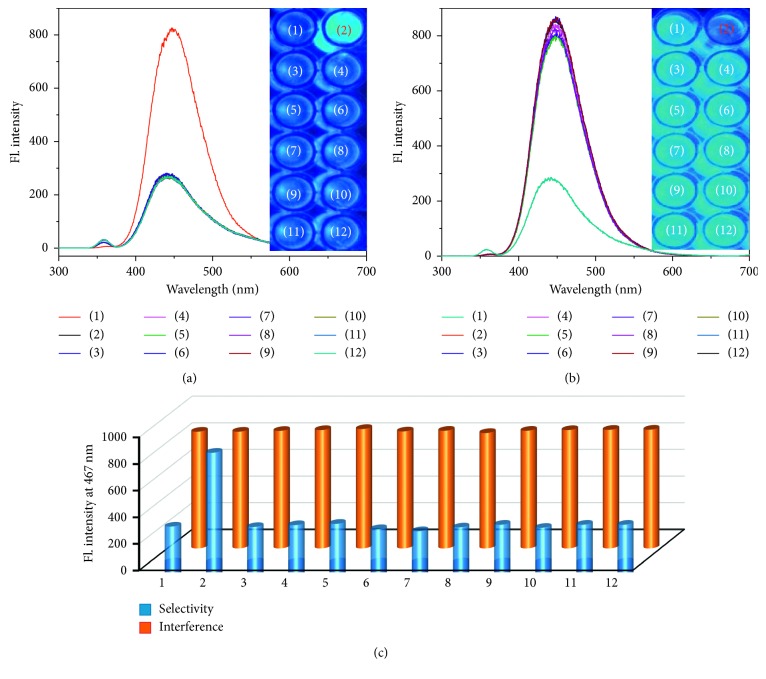
Fluorescence emission spectra of TPIOP boronate after reacting with (a) 15 *μ*M H_2_O_2_ and 15 mM of other ROS and (b) 15 *μ*M H_2_O_2_ along with 15 mM of other ROS. (c) Bar chart of selectivity and interferences of TPIOP boronate. Experimental conditions: 2 *μ*M of TPIOP boronate in 10 mM HEPES buffer solution (pH 7.4 and 2 vol% DMSO). (1) TPIOP probe, (2) H_2_O_2_, (3) KO_2_, (4) NO_3_^−^, (5) TBHP, (6) mCPBA, (7) HOCl, (8) ClO_4_^−^, (9) SO_4_^2−^, (10) NO_2_^−^, (11) ONOO^−^, and (12) BPO, respectively. Insets: corresponding fluorescence images.

Further investigations were carried out to determine the selectivity and inference ability of TPIOP boronate toward H_2_O_2_. In addition, the fluorescence response of TPIOP boronate toward H_2_O_2_ was assayed for various ROS. The fluorescence response of TPIOP boronate upon adding 15 *μ*M H_2_O_2_ to it is illustrated in Figure 5. Under similar reaction conditions, adding 15 mM solutions (1000× more concentrated) of other ROS, such as KO_2_, NO_3_^−^, TBHP, mCPBA, HOCl, ClO_4_^−^, SO_4_^2−^, and NO_2_^−^, to TPIOP boronate did not induce significant changes in the fluorescence intensity compared to that of H_2_O_2_ (Figure 5(a)), and the detection of H_2_O_2_ was not hindered even in the presence of ROS solutions 1000× more concentrated than the H_2_O_2_ solution (Figure 5(b)). These findings were also confirmed by the fluorescence images in the insets of Figures 5(a) and 5(b) and indicated that TPIOP boronate can also be used to visually identify and discriminate the presence of H_2_O_2_ from wide ranges of ROS pools under UV radiation. The bar chart representation of the fluorescence response of TPIOP boronate in the presence of various ROS is shown in Figure 5(c). From the above studies, we concluded that TPIOP boronate was 1000 times more sensitive to H_2_O_2_ than to other ROS when other ROS were present. Also, it was clearly observed that the TPIOP boronate probe presented rapid and nanomolar level sensitivity toward H_2_O_2_.

These results obtained using the TPIOP boronate encouraged us to further investigate the possibility of using it for fluorescence imaging in living cells. Before considering the biological applications of TPIOP boronate, its sensitivity in terms of its fluorescent behavior was tested as a function of pH. The fluorescence emission intensity at 467 nm was monitored before and after adding 15 *μ*M H_2_O_2_ to a 2 *μ*M TPIOP boronate probe solution and was plotted against the pH (Figure S7A). The fluorescence images observed for TPIOP boronate at different pH levels and the fluorescence responses after adding H_2_O_2_ to it are illustrated in Figure S7B. It can be noticed that the emission intensity is usually high in basic media (pH 7–12), but it reached its maximum at pH 7-8. Hence, the probe solution was maintained at the physiological pH of 7.4 throughout all the experiments.

### 3.5. Cytotoxicity of TPIOP Boronate and Its H_2_O_2_ Detection in Live MCF-7 Cells

**Figure 6:**
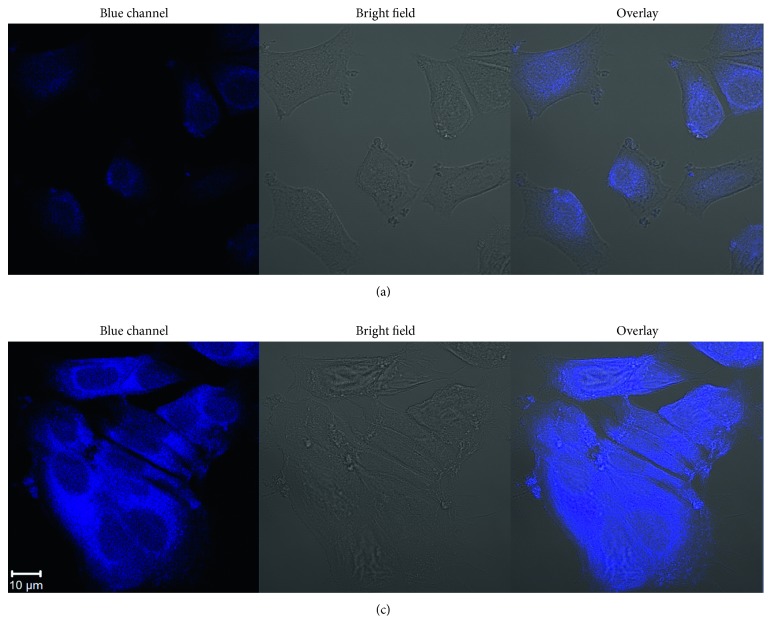
Confocal fluorescence microscopy images of MCF-7 cells incubated with (a) 10 *μ*M probe and (b) 10 *μ*M H_2_O_2_ and probe. Scale bar = 10 *μ*m (blue channel of (b)).

Since the TPIOP boronate probe exhibited excellent sensitivity (nM level) for the detection of H_2_O_2_, we hypothesized that it would be possible to detect H_2_O_2_ in cell lines using TPIOP boronate. Before using the TPIOP boronate probe for live cell imaging, its biocompatibility was tested using an MTT assay. The bioimaging applications of TPIOP boronate for detecting H_2_O_2_ were demonstrated using living MCF-7 cells. The cytotoxicity of the probe was low, as shown in Figure S8, which reveals that the MCF-7 cells were able to survive concentration of TPIOP boronate up to 100 *μ*M. We chose a 10 *μ*M solution of TPIOP boronate for staining the probe. Living MCF-7 cells stained with TPIOP boronate were used to detect H_2_O_2_, and live images were recorded using a Zeiss LSM 700 confocal microscope. Images of H_2_O_2_ in the MCF-7 cells were recorded after the TPIOP boronate probe (10 *μ*M) and 10 *μ*M H_2_O_2_ were incubated for 30 min at 37°C. The TPIOP boronate-stained MCF-7 cells generated weak blue emissions (Figure 6(a)), whereas the TPIOP boronate-stained MCF-7 cells incubated with H_2_O_2_ generated strong blue emissions (Figure 6(b)). The increase in the emission intensity of the probe after interacting with the live MCF-7 cells was due to H_2_O_2_ and is illustrated in Figure S9. These results demonstrated that the TPIOP boronate probe in our study would be amenable for live-cell H_2_O_2_ imaging.

## 4. Conclusions

The findings of our current study indicated that a novel TPIOP boronate chemodosimeter containing highly polar C–B bond was designed, synthesized, characterized, and utilized for H_2_O_2_ detection. Increasing the polarity of the C–B bond can increase the reactivity of TPIOP boronate with H_2_O_2_. This was achieved by introducing a TPIOP moiety using CPB. Adding H_2_O_2_ to TPIOP boronate triggered the formation of TPIOP-OH, and then the fluorescence intensity was increased via the AIE mechanism. Therefore, the abovementioned TPIOP boronate probe could become a fluorescent tool for the rapid, selective, and sensitive monitoring of H_2_O_2_. The lowest LOD of 8 nM was achieved using TPIOP boronate at physiological pH level, which was below the range reported in the literature. In addition, paper strips coated with TPIOP boronate were used for on-site naked-eye H_2_O_2_ detecting experiments using UV radiation. Furthermore, TPIOP boronate exhibited low cytotoxicity and was utilized as fluorescent marker for detecting H_2_O_2_ in living cells [35].

## Figures and Tables

**Table 1:** Comparison of LOD and response time values of present probe with those of boronate-based H_2_O_2_ sensors recently reported in the literature.

No.	System	Response time (min)	LOD (nM)	References
1	Boronic ester of TPE	∼10	520	[5]
2	Carbon dot featuring boronate recognition unit	30	750	[7]
3	*N*-4-(Benzyl boronic pinacol ester) pyridinium bromide moiety on TPE	30	180	[8]
4	TPE modified with boronic ester	10	950	[9]
5	Diboronate functionalized TPE	60	3200	[10]
6	Imine derivative of TPE	40	100	[11]
7	Modified hemicyanine dye	90	13	[12]
8	9H-1,3-dichloro-7-hydroxy-9,9-dimethylacridine-2-one	60	420	[13]
9	2-(3-(4-Hydroxystyryl)-5,5-dimethylcyclohex-2-enylidene) malononitrile	30	70	[14]
10	Mitochondria targeted benzothiazole probe	120	23	[15]
11	Phenanthroimidazole, benzonitrile, and phenyl boronate	10	148	[16]
12	4-Carboxy-3-fluorophenylboronic acid and 7-hydroxycoumarin-conjugated probe	60	760	[17]
13	1,3-Bis(bispyridin-2-ylimino)isoindolin-4-ol	40	9.1	[18]
14	Tetrahydroquinoxaline iminocoumarin derivative	10	60	[19]
15	Dioxetane-based probe	—	75000	[20]
16	2-Dicyanomethylene-3-cyano-4,5,5-trimethyl-2,5-dihydrofuran	60	61	[21]
17	Naphthalimide-coumarin-based ICT-activated FRET sensor	60	1350	[22]
18	Lysosome-targeted two-photon probe	—	1210	[23]
19	Cyanosilbene-boronate-based AIEE probe	—	455	[24]
20	Borylated boron dibenzopyrromethene dye	—	248	[25]
21	Mitochondria-targeted probe	4	40	[26]
22	*p*-Nitroaniline and 7-amino-4-methyl coumarin bearing probe	8	100	[27]
23	Dicyanomethylene-4-H-chromene	10	450	[28]
24	Dicyanomethylene-4-H-pyran	30	79	[29]
25	4-Hydroxynaphthalimide derivate	40	2000	[30]
26	TPIOP boronate probe in this study	2	8	This work

## Data Availability

The data used to support the findings of this study are included within the article and the supplementary information file(s).

## Supplementary Materials

Supplementary Materials Figure S1: 1H NMR spectra of (A) TPIOP boronate and (B) TPIOP-OH. Figure S2: 13C NMR spectra of (A) TPIOP boronate and (B) TPIOP-OH. Figure S3: HR-MS of (A) TPIOP boronate and (B) TPIOP-OH. Figure S4: molecular model of TPIOP boronate and its atomic charges. Figure S5: electrostatic potential diagram of TPIOP boronate. Figure S6: UV-Vis spectra for different concentration of H_2_O_2_ in (A) 20 *μ*M TPIOP boronate and (B) its corresponding linearity plot. Figure S7: (A) fluorescence intensity at 467 nm for 2 *μ*M TPIOP boronate before and after reacting with H_2_O_2_ and (B) fluorescence images obtained for 2 *μ*M TPIOP boronate. Figure S8: cytotoxicity test results of MTT assay. Figure S9: fluorescence intensity change (A) before (B) after adding H_2_O_2_ to TPIOP boronate stained live MCF-7 cell lines.

## References

[1] Atreaux B. D., Toledano M. B. (2007). ROS as signaling molecules mechanisms that generate specificity in ROS homeostasis. *Nature Reviews Molecular Cell Biology*.

[2] Finkel T. (2003). Oxidant signals and oxidative stress. *Current Opinion in Cell Biology*.

[3] Lacina K., Skladal P., James T. D. (2014). Boronic acids for sensing and other applications-a mini-review of papers published in 2013. *Chemistry Central Journal*.

[4] Lippert A. R., Van de Bittner G. C., Chang C. J. (2011). Boronate oxidation as a bioorthogonal reaction approach for studying the chemistry of hydrogen peroxide in living systems. *Accounts of Chemical Research*.

[5] Zhang W., Liu W., Li P., Huang F., Wang H., Tang B. (2015). Rapid-response fluorescent probe for hydrogen peroxide in living cells based on increased polarity of C–B bonds. *Analytical Chemistry*.

[6] Han J., Chu C., Cao G. (2018). A simple boronic acid-based fluorescent probe for selective detection of hydrogen peroxide in solutions and living cells. *Bioorganic Chemistry*.

[7] Du F., Min Y., Zeng F., Yu C., Wu S. (2014). A targeted and FRET-based ratiometric fluorescent nanoprobe for imaging mitochondrial hydrogen peroxide in living cells. *Small*.

[8] Hu F., Huang Y., Zhang G., Zhao R., Zhang D. (2014). A highly selective fluorescence turn-on detection of hydrogen peroxide and d-glucose based on the aggregation/deaggregation of a modified tetraphenylethylene. *Tetrahedron Letters*.

[9] Qiao J., Liu Z., Tian Y., Wu M., Niu Z. (2015). Multifunctional self-assembled polymeric nanoprobes for FRET-based ratiometric detection of mitochondrial H_2_O_2_ in living cells. *Chemical Communications*.

[10] Liu G.-J., Long Z., Lv H.-j., Li C.-y., Xing G.-w. (2016). A dialdehyde-diboronate-functionalized AIE luminogen: design, synthesis and application in the detection of hydrogen peroxide. *Chemical Communications*.

[11] Song Z., Kwok R. T. K., Ding D. (2016). An AIE-active fluorescence turn-on bioprobe mediated by hydrogen-bonding interaction for highly sensitive detection of hydrogen peroxide and glucose. *Chemical Communications*.

[12] Xu F., Li H., Yao Q., Fan J., Wang J., Peng X. (2016). A NIR fluorescent probe: imaging endogenous hydrogen peroxide during an autophagy process induced by rapamycin. *Journal of Materials Chemistry B*.

[13] Hou J., Qian M., Zhao H. (2018). A near-infrared ratiometric/turn-on fluorescent probe for in vivo imaging of hydrogen peroxide in a murine model of acute inflammation. *Analytica Chimica Acta*.

[14] Zhang L., Qian M., Xia J. (2019). A NIR fluorescent sensor with large Stokes shift for the real-time visualization of endogenous hydrogen peroxide in living cells. *Journal of Photochemistry and Photobiology A: Chemistry*.

[15] He L., Liu X., Zhang Y. (2018). A mitochondria-targeting ratiometric fluorescent probe for imaging hydrogen peroxide with long-wavelength emission and large stokes shift. *Sensors and Actuators B: Chemical*.

[16] Chen Y., Shi X., Lu Z., Wang X., Wang Z. (2017). A fluorescent probe for hydrogen peroxide in vivo based on the modulation of intramolecular charge transfer. *Analytical Chemistry*.

[17] Feng C., Wang F., Dang Y., Xu Z., Yu H., Zhang W. (2017). A self-assembled ratiometric polymeric nanoprobe for highly selective fluorescence detection of hydrogen peroxide. *Langmuir*.

[18] Liu X., Tian H., Yang L., Su Y., Guo M., Song X. (2017). A sensitive and selective fluorescent probe for the detection of hydrogen peroxide with a red emission and a large stokes shift. *Sensors and Actuators B: Chemical*.

[19] Liu X., He L., Yang L., Geng Y., Yang L., Song X. (2018). Iminocoumarin-based fluorescence probe for intracellular H_2_O_2_ detection with a red emission and a large stokes shift. *Sensors and Actuators B: Chemical*.

[20] Seven O., Sozmen F., Simsek Turan I. (2017). Self immolative dioxetane based chemiluminescent probe for H_2_O_2_ detection. *Sensors and Actuators B: Chemical*.

[21] Xu F., Tang W., Kang S., Song J., Duan X. (2018). A highly sensitive and photo-stable fluorescent probe for endogenous intracellular H_2_O_2_ imaging in live cancer cells. *Dyes and Pigments*.

[22] Xu K., He L., Yang X., Yang Y., Lin W. (2018). A ratiometric fluorescent hydrogen peroxide chemosensor manipulated by an ICT-activated FRET mechanism and its bioimaging application in living cells and zebrafish. *The Analyst*.

[23] Ren M., Deng B., Wang J.-Y. (2016). A fast responsive two-photon fluorescent probe for imaging H_2_O_2_ in lysosomes with a large turn-on fluorescence signal. *Biosensors and Bioelectronics*.

[24] Dhoun S., Kaur S., Kaur P., Singh K. (2017). A cyanostilbene-boronate based AIEE probe for hydrogen peroxide-application in chemical processing. *Sensors and Actuators B: Chemical*.

[25] Matsumoto A., Nishiyabu R., Kubo Y. (2014). Synthesis of a borylated boron-dibenzopyrromethene dye enabling the visual detection of H_2_O_2_ vapor. *RSC Advances.*.

[26] Xu J., Zhang Y., Yu H., Gao X., Shao S. (2016). Mitochondria-targeted fluorescent probe for imaging hydrogen peroxide in living cells. *Analytical Chemistry*.

[27] Lo L.-C., Chu C.-Y. (2003). Development of highly selective and sensitive probes for hydrogen peroxide electronic supplementary information (ESI) available: general methods. See http://www.rsc.org/suppdata/cc/b3/b309393j/. *Chemical Communications*.

[28] Wang P., Wang K., Gu Y. (2016). A highly selective fluorescent turn-on NIR probe for the bioimaging of hydrogen peroxide in vitro and in vivo. *Sensors and Actuators B: Chemical*.

[29] Wang P., Wang K., Chen D., Mao Y., Gu Y. (2015). A novel colorimetric and near-infrared fluorescent probe for hydrogen peroxide imaging in vitro and in vivo. *RSC Advances*.

[30] Zhu B., Jiang H., Guo B. (2013). A highly selective ratiometric fluorescent probe for hydrogen peroxide displaying a large emission shift. *Sensors and Actuators B: Chemical*.

[31] Hong Y., Lam J. W. Y., Tang B. Z. (2011). Aggregation-induced emission. *Chemical Society Reviews*.

[32] Salerno L., Pittalà V., Romeo G. (2015). Novel imidazole derivatives as heme oxygenase-1 (HO-1) and heme oxygenase-2 (HO-2) inhibitors and their cytotoxic activity in human-derived cancer cell lines. *European Journal of Medicinal Chemistry*.

[33] Takagi K., Kusafuka K., Ito Y. (2015). Synthesis and optical properties of imidazole- and benzimidazole-based fused *π*-conjugated compounds: influence of substituent, counteranion, and *π*-conjugated system. *Journal of Organic Chemistry*.

[34] Wu C.-W., Tsai C.-M., Lin H.-C. (2006). Synthesis and characterization of poly(fluorene)-based copolymers containing various 1,3,4-oxadiazole dendritic pendants. *Macromolecules*.

[35] Lee C., Yang W., Parr R. G. (1988). Development of the Colle-Salvetti correlation-energy formula into a functional of the electron density. *Physical Review B*.

[36] Krishnan R., Binkley J. S., Seeger R., Pople J. A. (1980). Self-consistent molecular orbital methods. XX. A basis set for correlated wave functions. *Journal of Chemical Physics*.

[37] Shao Y. (2015). Advances in molecular quantum chemistry contained in the Q-chem 4 program package. *Molecular Physics*.

[38] Rajalakshmi K., Nam Y.-S., Selvaraj M., Lee Y., Lee K.-B. (2018). Metal free bioimaging reagent for intracellular citrate in prostate cancer cells using aryl boronate derivative. *Sensors and Actuators B: Chemical*.

