# Supplementary material for: Rapid-Response and Highly Sensitive Boronate Derivative-Based Fluorescence Probe for Detecting H2O2 in Living Cells
Source: J Anal Methods Chem. 2019 May 2;2019:5174764. doi: 10.1155/2019/5174764 (PMC6525842; doi:10.1155/2019/5174764)
Supplement: Supplementary Materials — Figure S1: 1H NMR spectra of (A) TPIOP boronate and (B) TPIOP-OH. Figure S2: 13C NMR spectra of (A) TPIOP boronate and (B) TPIOP-OH. Figure S3: HR-MS of (A) TPIOP boronate and (B) TPIOP-OH. Figure S4: molecular model of TPIOP boronate and its atomic charges. Figure S5: electrostatic potential diagram of TPIOP boronate. Figure S6: UV-Vis spectra for different concentration of H2O2 in (A) 20 μM TPIOP boronate and (B) its corresponding linearity plot. Figure S7: (A) fluorescence intensity at 467 nm for 2 μM TPIOP boronate before and after reacting with H2O2 and (B) fluorescence images obtained for 2 μM TPIOP boronate. Figure S8: cytotoxicity test results of MTT assay. Figure S9: fluorescence intensity change (A) before (B) after adding H2O2 to TPIOP boronate stained live MCF-7 cell lines. [file 5174764.f1.pdf]

Supplementary Material

Rapid-response and highly sensitive boronate derivative-based fluorescence probe for detecting H_2O_2 in living cells

Muthusamy Selvaraj,[1] Kanagaraj Rajalakshmi,[1] Yun-Sik Nam,[2] Yeonhee Lee,[2] Byoung-Chan Kim,[1] Sung Jin Pai,[3] Sang Soo Han,[3] and Kang-Bong Lee[1]

[1]Green City Technology Institute, [2]Advanced Analysis Center, and [3]Computational Science Center, Korea Institute of Science & Technology, Hwarang-ro 14-gil 5 Seongbuk-gu, Seoul 02792, Republic of Korea

*Corresponding author. Tel.: +82 2 958 5957; fax.: +82 2 958 5810

E-mail address: leekb@kist.re.kr (K.-B. Lee).

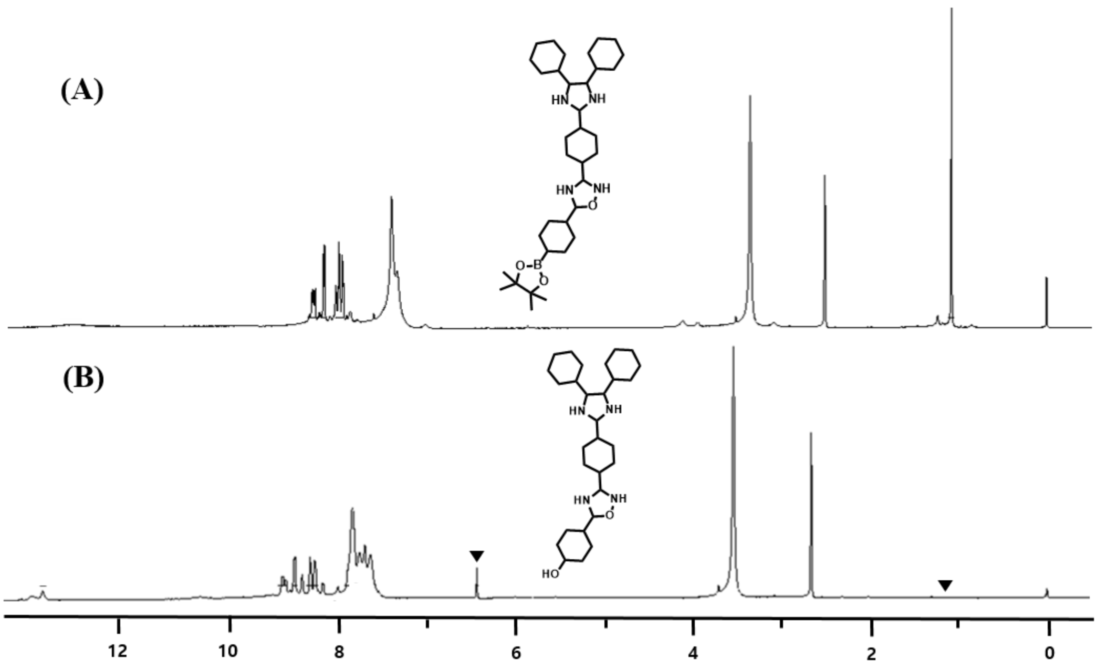

FIGURE S1: ^{1}H NMR spectra of (A) TPIOP boronate and (B) TPIOP-OH.

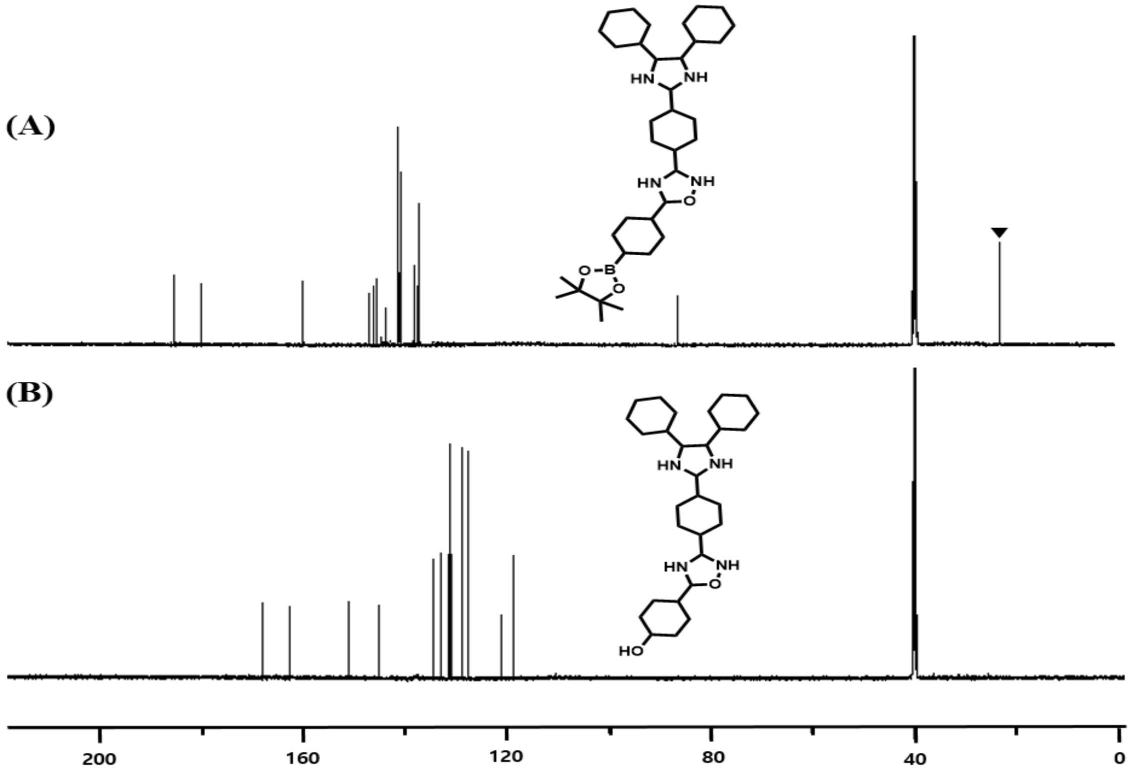

FIGURE S2: ^{13}C NMR spectra of (A) TPIOP boronate and (B) TPIOP-OH.

(A) (B)

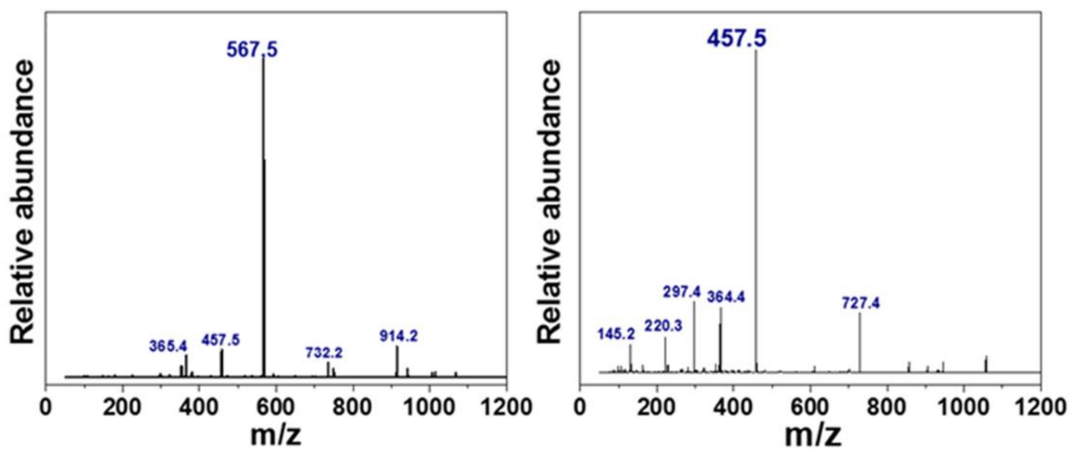

FIGURE S3: HR-MS of (A) TPIOP boronate and (B) TPIOP-OH.

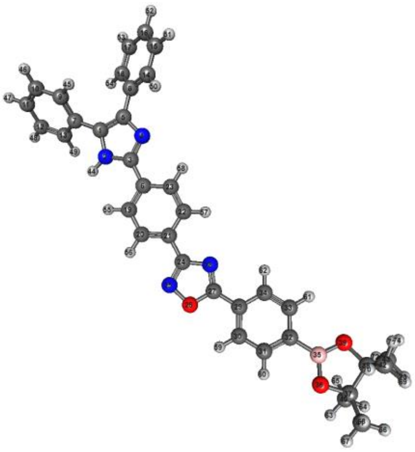

Atom	No	Charge
C	1	0.13048
N	2	-0.53908
C	3	0.37115
N	4	-0.5221
C	5	0.11324
C	6	-0.09175
C	7	-0.084
C	8	-0.07227
C	9	-0.22692
C	10	-0.24996
C	11	-0.25084
C	12	-0.24848
C	13	-0.23786
C	14	-0.23485
C	15	-0.25296
C	16	-0.25737
C	17	-0.25328
C	18	-0.23567
C	19	-0.2313
C	20	-0.1928
C	21	-0.10924
C	22	-0.21314
C	23	-0.20582
C	24	0.34415
N	25	-0.19475
O	26	-0.33086
C	27	0.57414
N	28	-0.52729
C	29	-0.12925
C	30	-0.19874
C	31	-0.20468
**C**	**32**	**-0.3863**
C	33	-0.20556
C	34	-0.19226
**B**	**35**	**1.15443**
**O**	**36**	**-0.75739**
C	37	0.23837
C	38	0.23823
**O**	**39**	**-0.75673**
C	40	-0.7185
C	41	-0.71301
C	42	-0.71869
C	43	-0.71313
H	44	0.48103
H	45	0.26311
H	46	0.2616
H	47	0.26052
H	48	0.26175
H	49	0.26127
H	50	0.25985
H	51	0.25888
H	52	0.25825
H	53	0.25909
H	54	0.2591
H	55	0.26224
H	56	0.26839
H	57	0.27156
H	58	0.26869
H	59	0.27005
H	60	0.26548
H	61	0.26534
H	62	0.27033
H	63	0.25641
H	64	0.25683
H	65	0.25102
H	66	0.25841
H	67	0.25535
H	68	0.25469
H	69	0.25706
H	70	0.25095
H	71	0.25666
H	72	0.25511
H	73	0.25848
H	74	0.25514

FIGURE S4: Molecular model of TPIOP boronate and its atomic charges.

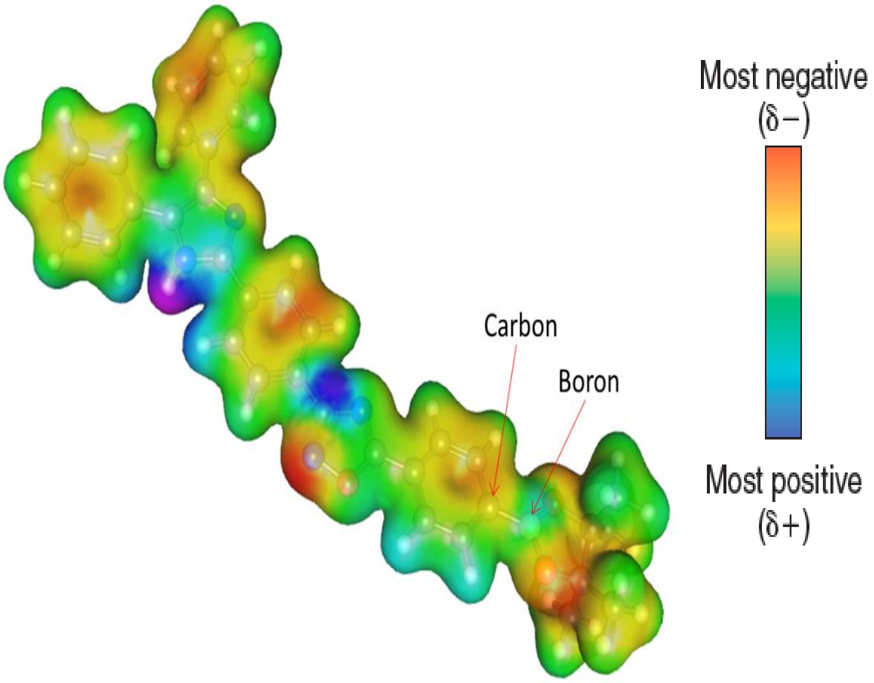

FIGURE S5: Electrostatic potential diagram of TPIOP boronate.

(A) (B)

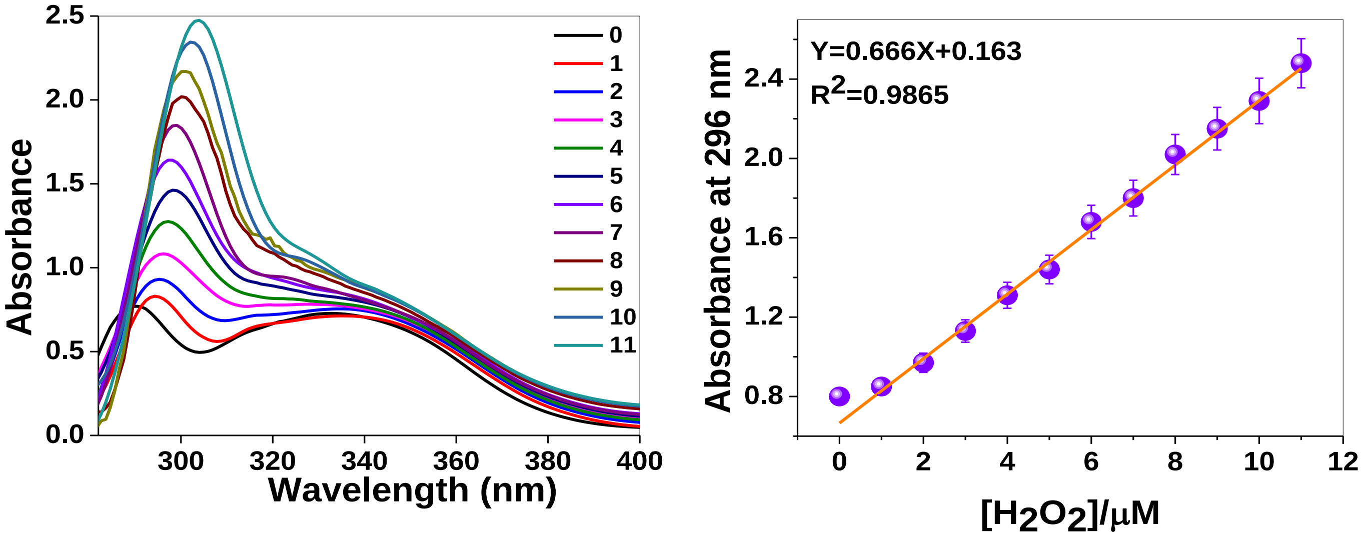

FIGURE S6: UV-Vis spectra for different concentration (0 to 11 µM) of H_2O_2 in (A) 20 µM TPIOP boronate in 10 mM HEPES buffer solution (pH 7.4, and 2 vol% DMSO) and (B) its corresponding linearity plot.

(A)

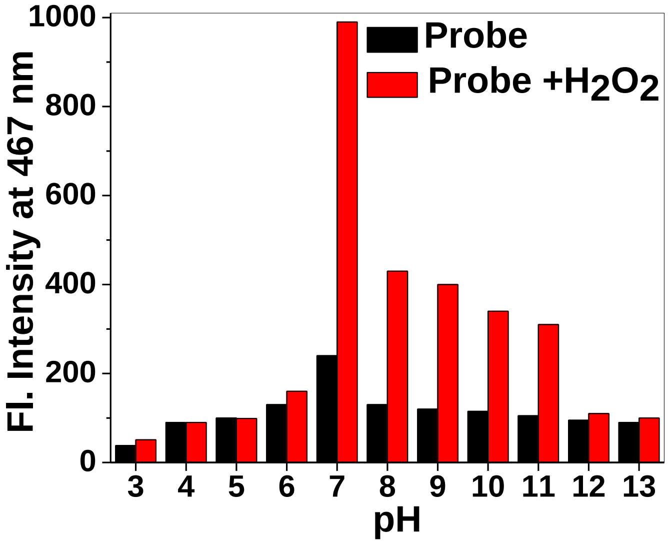

(B)

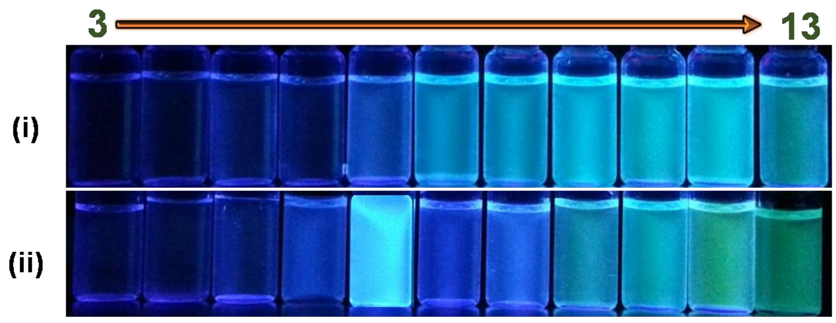

FIGURE S7: (A) Fluorescence intensity at 467 nm for 2 µM TPIOP boronate before and after reacting with 15 µM H_2O_2 at various pH levels (3 to 13). (B) Fluorescence images obtained for 2 µM TPIOP boronate at different pH levels (3 to 13) (i) before and (ii) after reacting with 15 µM H_2O_2.

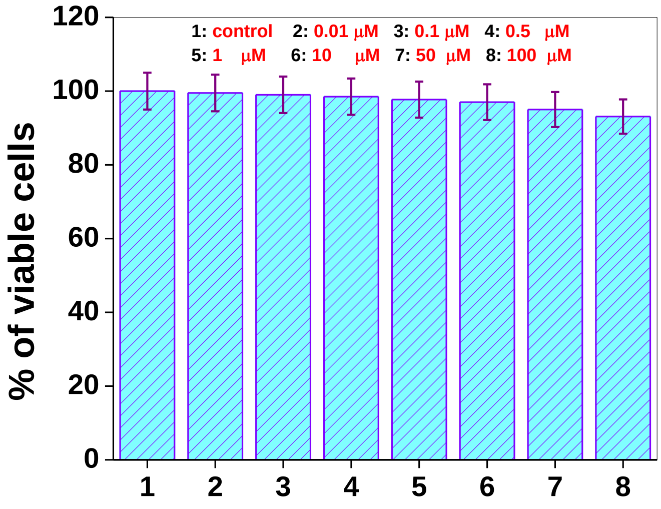

FIGURE S8: Cytotoxicity test results of MTT assay. The percentages of viable cells are plotted on the y axis. (Average of 5 replicates).

(A)

(B)

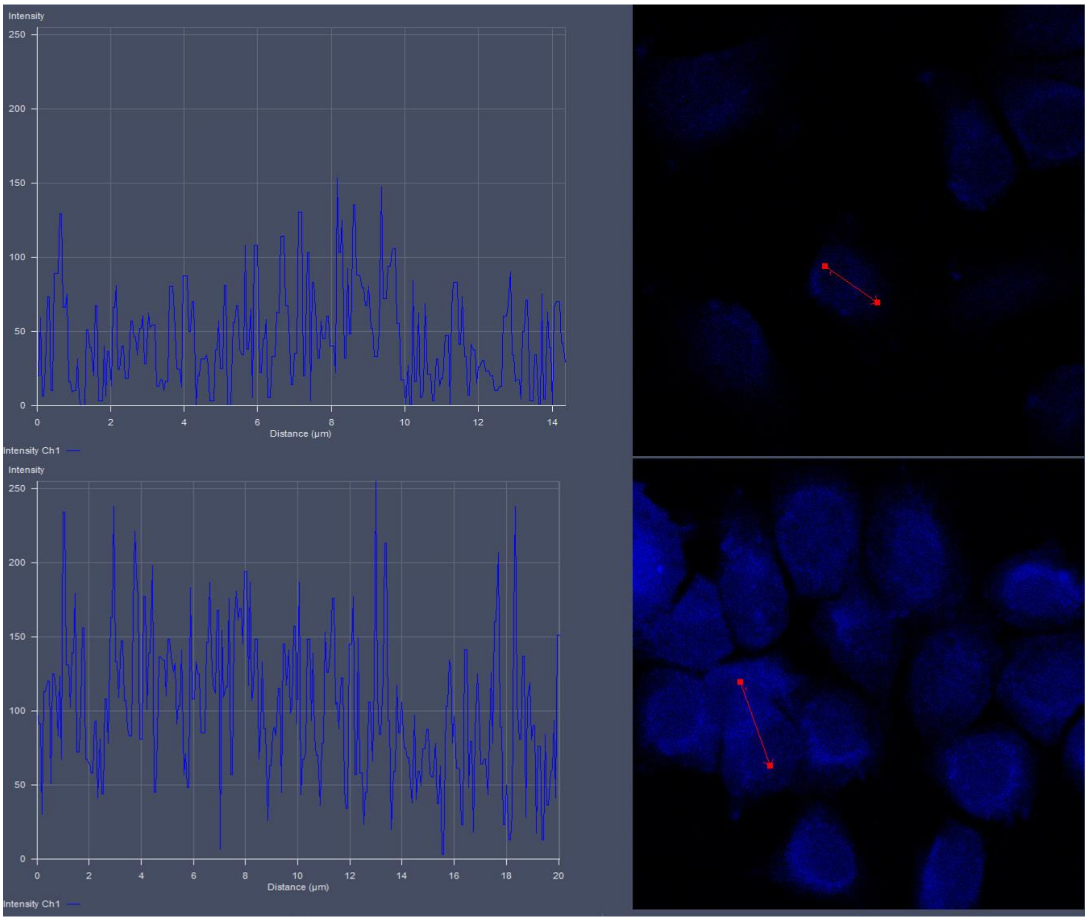

FIGURE S9: Fluorescence intensity change (A) before (B) after adding H_2O_2 to TPIOP boronate stained live MCF-7 cell lines.
